# Breaking the barriers of animosity: innovation in business models as a positioning strategy

**DOI:** 10.1016/j.heliyon.2021.e07545

**Published:** 2021-07-10

**Authors:** Jose Andres Areiza-Padilla, Mihaela Simona Moise, Mario Andres Manzi Puertas

**Affiliations:** a Department of Business Administration, Pontificia Universidad Javeriana, Bogotá 110231, Colombia; b Department of Marketing, University of Valencia, 46022 Valencia, Spain

**Keywords:** Business model innovations, Consumer animosity, Collective tendencies, Patriotic tendencies, Ethnocentrism tendencies, Foreign brand image

## Abstract

Consumer animosity is often studied in the large economies of the world, in order to explain the negative feelings generated by an individual towards another country and its products, due to various political, economic and social conflicts. This study presents three specific developments in this field. First, to demonstrate how companies in the retail sector have been able to develop innovations in their business models through their shops and virtual channels, which generate a positive positioning in the mind of the consumer, capable of minimizing animosity towards them. In this way it is shown that a consumer can have strong feelings of patriotism, animosity and ethnocentrism, and yet a positive image towards a brand or product of the country with which the conflict takes place. Second, a contribution to literature regarding the scant research on consumer animosity in developing countries, and specifically in Latin America. Third, an analysis of animosity under a current context of conflict between countries, and not of studies carried out taking situations or facts from the past. In this way, a contribution is generated that allows to understand more the behavior of the consumer and his animosity in societies with emerging economies and as the innovations in business models allow to improve both the economic profitability of a company, as its brand image.

## 1 Introduction

Throughout the history of humanity, various economic, political, religious and cultural conflicts between different societies have been documented; in this way, antagonism in human beings is a very ancient and deeply studied topic in classical ethnographies, which have highlighted the importance of antagonistic interactions between different groups and between their individuals (Isakov et al., 2019).

Bearing this in mind, animosity arises as one of the ways to explain the strong negative feelings that can be held against a foreign country, due to any type of previous hostility on the part of this country (Averill, 1983). Thus, just as a war between countries leads to the existence of a warlike animosity, a commercial disagreement between countries, leads to an economic animosity, which is studied through the concept of consumer animosity (Klein et al., 1998).

Consumer animosity then emerges, as a particular current in literature, that allows to examine the impact of the very negative feelings of individuals, towards a particular country, and in particular against products or services manufactured or offered in that country.

In this way, the feelings of hatred caused by adverse actions to a citizen, or against his organizations or government, are directly reflected in the perceptions that consumers have, on the products of the country that originates such attacks (Maher et al., 2010).

However, most previous studies on consumer animosity have focused on its application in the world's major economies (Nijssen and Douglas, 2004; Amine, 2008; Alvarez and Campo, 2020). We believe that this is perhaps due to the political, economic and military importance of these countries, compared to other developing or underdeveloped countries. For this reason it is very difficult to find similar studies applied in Latin America and the Caribbean. On the other hand, this study focuses on consumer animosity in the midst of a current conflict between two countries, and also studies the retail pharmaceutical industry. In view of the above, there is an academic motivation to be able to understand the implications of consumer animosity in this type of society and in this economic sector, in order to understand if there are greater differences with respect to the major economies of the world.

Taking into account the above, this research proposes a study of consumer animosity between two developing countries in South America (Colombia and Venezuela), and in this way, be able to contribute to the little literature that exists in developing countries. Colombia and Venezuela are bordering countries, which currently have great political differences, which have affected their bilateral relations over the past 20 years.

Previous sociological studies, such as those of Doocy et al. (2019) and Aliaga Sáez et al. (2020), have been able to document how the political, social and economic crisis in Venezuela has produced a massive exodus of more than three million Venezuelans to other countries of the world.

Colombia, as its largest land border, is the country with the largest number of Venezuelan immigrants in the world; according to Colombian migration data, by April 2020 there were 1,825,000 Venezuelans in Colombia, of whom 44% are legally and 56% illegally (Migración Colombia, 2020).

This large number of immigrants in Colombian territory has generated different types of social conflicts, bearing in mind that Colombia does not have a developed economy; therefore, it was not prepared for this type of large-scale social interactions; In this way, Colombia faces the greatest international immigration to its country in its entire history, further deepening its own social and economic problems (Aliaga Sáez et al. (2020); Martínez Moya, 2020).

On the other hand, the political relationship between both countries has been very deteriorated, where Colombians often hear direct attacks between the leaders of Colombia and Venezuela, and even a possible war between the two countries, due to the political differences between its rulers.

On the other hand, the brand image of a product or service whose country generates animosity is often negative on the part of the local consumer (Russell and Russell, 2010); however, in other studies such as those of Fakharmanesh and Ghanbarzade Miyandehi (2013), although they initially raised a negative relationship between the consumer's animosity and the image of a foreign product, their results were not significant between the two constructs, generating new questions regarding the behavior of animosity.

For this, our research analyzed the brand image of the chain of international pharmacy chain “Farmatodo” of Venezuelan origin that operates in Colombia since 2009 and that by 2020, had 59 stores in 6 cities in Colombia; below, we describe the innovation in business models that “Farmatodo” has made.

Before the arrival of “Farmatodo” in Colombia, there were only the typical traditional drugstores in the country with the exclusive sale of medicines. This concept changed completely through “Farmatodo” because its innovation in business models, included a novelty in the service, where in a single place could be obtained great variety of products in the categories of health, beauty, baby and personal care. This varied range of products allowed to meet different needs in one place.

On the other hand, the shopping experience offered face-to-face shops with very spacious premises and modern décor, unlike the traditional pharmacies that were characterized by being very small and old. In its premises is offered free of charge the intake of blood pressure, weight measurement and body mass index, glucose, injections and professional advice pharmaceutical and beauty.

For its international growth, “Farmatodo” established an agreement with Oracle Retail, which has allowed it to implement new stores more quickly and improve the agility of its internal processes, both in the in-person stores, as well as its website and home delivery.

Given the usual congestion that occurs in most pharmacies in the delivery of their online orders, “Farmatodo” has been able to position itself in the minds of consumers with a online orders of between 30 and 45 min, much less time than traditional pharmacies.

His innovation in business models was also based, on the knowledge of his inventory in real time and the triangulation of all its headquarters, to be able to meet the delivery times to the customer; in this way, they have been able to integrate information technology with their business processes through services such as Oracle Retail Merchandising System, Oracle Retail Store Inventory Management and Oracle Retail Warehouse Management System; which allows them to manage their business at a corporate level, and with Oracle Retail Xstore Point-of-Service they have been able to improve the consumer experience in stores.

For these reasons “Farmatodo” has positioned itself in the minds of Colombian consumers as the fastest chain in home delivery, and they also generate a service experience in their in-person stores; in this way, this research presents a contribution to the literature regarding the few investigations on animosity in developing countries, and specifically in Latin America.

For this research we propose as a novelty, an analysis of animosity under a current context of conflict between countries, and not based on past historical situations, where we want to demonstrate that a consumer can have strong feelings of patriotism, of animosity and ethnocentrism, and yet a positive image towards a foreign brand or product, originating in the country with which there is some conflict.

In other words, there is not always widespread consumer animosity, but it is focused on specific brands or products with which some conflict is generated. This is possible if this brand, although it is foreign, has roots in the local culture, and has managed to position itself positively among the consumer, since before animosity was generated. For this research, patriotism and collectivism have been proposed as antecedents of consumer animosity, and ethnocentrism and image as results.

## 2 The conceptual framework: literature review and development of the hypotheses

### 2.1 Animosity and consumer animosity

Anonymity refers to strong feelings of disgust and enmity, which arise from previous or current hostilities, whether military, political, economic, social, etc. between different peoples and nations, especially towards those nations that are perceived as inciting, or violating social norms (Averill, 1983). In this way animosity can be classified according to the duration of the negative feeling towards another country:

Situational animosity refers to strong feelings of enmity that are associated with a specific circumstance, but that may be transient; on the other hand, stable animosity refers to antagonistic emotions that are accumulated over the years, so this stable animosity is very long-lasting and deeply rooted in people's minds.

It is important to emphasize that, in order to feel animosity, one has not necessarily previously had a personal experience, but this negative experience can be transmitted from generation to generation through various mechanisms such as history books or through narratives of the people who did experience such harassment directly (Jung et al., 2002). Brummett et al. (1998), defined animosity through three components; a cognitive component, involving cynical beliefs and distrust of others, an attitudinal component, involving negative emotions of anger, contempt and disgust, and a behavioral component, which generates hostility through different behaviors that generate aggressive verbal or physical actions.

All these components are expressed towards a specific country, and not towards foreign countries in general, because it is based on very specific situations that generated this discomfort with some specific country. For Klein at al. (1998), the anger that can be felt towards a foreign country, can lead to products manufactured in that country, generate negative feelings against them also; in this way an animosity of the consumer is generated. This occasional animosity is related to the decision not to buy products made in the country with which there is some conflict, but not to the quality judgments of that product. By way of summary, we can find several approximations to the definition of the concept of consumer animosity, in the literature review in Table 1.

**Table 1:** Approximations to the and consumer animosity.

Author(s) and Year	Definition
(Klein et al., 1998 p.90)	“The remnants of antipathy related to previous or ongoing military, political, or economic events will affect consumers' purchase behavior in the international marketplace”
(Rose et al., 2009 p.330)	“Consumer animosity refers to strong negative emotions toward purchasing products from a disliked nation or group”
(Hoffmann et al., 2011 p.236)	“Consumer animosity is the antipathy toward a certain country, which negatively affects the intention to buy products imported from that country”
(Little and Singh, 2015 p.84)	“Antipathy toward nations, regions, cultures, groups, or organizations”
(Li et al., 2021 p.2)	“Critical factor affecting consumers' product perceptions and purchase behaviors, particularly in the context of international businesses”

Source: Author's own compilation.

### 2.2 Antecedents of consumer animosity

#### 2.2.1 Collective tendencies

In collectivist societies, individuals are integrated through strong ties that generate loyalty to their group, and for this reason they often place the interests of their group above their particular interests, thus maintaining a group solidarity (Klasing, 2013). For Greif (1994), in these partnerships, its members often feel very involved in the life of the other members of the group, in addition they do not usually cooperate between members of different groups. Their social structure is segregated to interact preferably with members of their own ethnic, religious, or family group. These collectivist values are usually present from the birth of a person, who is born and grows within a primary social group such as the family (Triandis, 2018).

Bearing this in mind, in collectivist cultures both the behaviour of its members and the behaviour of the group is governed by the interests of the same group, for this reason if there is some kind of conflict between the objectives of the group and the personal objectives of the individual, this difference is resolved in favor of the interests of the group (Triandis et al., 1990).

For Han (2017), collectivism is one of the basic dimensions that explain the thoughts, perceptions, actions, behaviors and value system among the members of a society. In this way collectivism is a link between individuals who see themselves as part of a group and for this reason, this influences the way the individual makes his decisions.

Based on the concept of collectivism and previous studies by Han (2017); Latif et al. (2019), for this study we consider that societies with high values of collectivism will react very closely to possible external threats, In this way, we formulate the following hypotheses:

Hypothesis 1 (H1). Collective tendencies in colombian society have a positive impact on consumer animosity

#### 2.2.2 Patriotic tendencies

For Sumner (1906) patriotism is loyalty to the group to which one belongs by birth and which allows to generate feelings of companionship and cooperation, towards all the hopes and sufferings of the group. On the other hand, the sociologist Durkheim (2018), defined patriotism as a type of religion, and therefore a kind of cult towards the State, in which its citizens worship it; generating a set of ideas and feelings that unite a person with his State, and therefore his loyalty to this. In this way each nation has its own characteristics so that its citizens feel proud of this; in addition it could be compatible with the search for a more fair world for all, instead of being a source of domination towards other societies (Durkheim, 2018). On these patriotic trends and values, Van der Toorn et al. (2014) realize a clear difference between the values of patriotism and nationalism, which may exist in a society; although both terms are related as a form of national identification, are different concepts.

Nationalism implies a superiority and domination of one country over others, without there being some kind of internal criticism, and where the social values of other countries are devalued; patriotism, on the other hand, is a feeling of love for one's country, without implying domination over others, it also implies an internal comparison of the quality of life of its citizens with that of other nations, which even allows an internal criticism if necessary.

Taking into account the positive feelings that patriotic societies generate towards the country of origin, to the concerns that these citizens may feel for an external threat, and based on previous studies of Klein and Ettensoe (1999), Hoffmann et al. (2011), Yang et al. (2015), Al Ganideh and Elahee (2018), which found a positive relationship between patriotism and animosity; we formulate the following Hypothesis:

Hypothesis 2 (H2). Patriotic tendencies of colombian society have a positive impact on consumer animosity

On the other hand, taking into account the previous definitions of collectivism and patriotism, for this study we consider that societies with high values of collectivism generate actions that foster social cohesion among its members and exalt their levels of patriotism as a sign of pride, loyalty and love for their own country. For this reason, and based on previous studies by Realo, and Allik (1999); Sagy et al. (2001); Anthony et al. (2003); Steele and Lynch (2013), we formulated the following Hypothesis:

Hypothesis 3 (H3). Collective tendencies in Colombian society have a positive impact on their patriotic tendencies

### 2.3 The results of consumer animosity

#### 2.3.1 Ethnocentric tendencies

For Schopmeyer and Fisher, 1993, the study of the concept of ethnocentrism is usually common in sociology courses, to sensitize students on how some ways of thinking are shaped through different perceptions, influences and social judgments. These influences range from internal groups to external groups; as ethnocentrism often leads to a double judgment of cultural patterns. On the one hand, these patterns consider normal any kind of practices of the individual's own society, which are accepted uncritically; however, it generates at the same time a rejection and intolerance towards the practices of other societies, which are considered abnormal (Schopmeyer and Fisher, 1993).

Sumner (1906) defined ethnocentrism as the perception of individuals towards the group to which they belong, and the valuation of that group, with respect to other distinct groups; in this way, the perception of these individuals about their own group becomes their point of reference towards everything, and everything else is valued and ranked with reference to this. For this reason, each group will exalt its superiority and despise the outer groups.

In this way, groups that have a strong ethnocentric tendency end up valuing as inferior everything that does not belong to their group, in addition, they acquire an attitude towards them of suspicion and hostility (Sumner, 1906). Bizumic (2018), defined ethnocentrism as an attitudinal construct that has strong cognitive aspects (beliefs), as well as affective (emotions), and aspects of behavior (actions). This construct is rooted in the human mind and is found in all ethnic groups, albeit to varying degrees. In this way, one can understand the selfishness of one ethnic group towards other groups, in any type of society (industrialized and non-industrialized), which often feel superior, better and more important than others, because ethnocentrism is both a social and psychological construction that is related to the belief of people of their own ethnic group, they are the center of everything (Bizumic, 2018).

Taking into account the negative feelings that ethnocentrism usually generates towards the purchase of foreign products (Baruk, 2019), and previous studies by Nakos and Hajidimitriou (2007), Cheah et al. (2016), Lee et al. (2017), Souiden et al. (2018), where ethnocentrism is positively related to animosity, we formulate the following Hypothesis:

Hypothesis 4 (H4). Consumer animosity has a positive impact on ethnocentric tendencies

#### 2.3.2 Image

The image of a brand is a mental scheme formed by a network of concepts or nodes that are interconnected by different links and associations (Salinas and Pérez, 2009). These associations of the brand image are multidimensional, and have both an affective and a perceived quality dimension, in this way it is defined as the sum of all the associations kept in the memory of a person, which lead to a perception of it (Keller 1993).

From the sociological point of view, Roth's research, (1995), determined that in societies that possess high values of individualism, the brand image should emphasize its functionality, and its novelty, in addition to the experiences that are transmitted through them. On the other hand, in societies with high collectivism values the brand image should emphasize the benefits of membership and group membership.

Some previous studies such as those by Russell and Russell (2010) determined a negative relationship between consumer animosity and image towards foreign products, however, other studies such as those by, Fakharmanesh and Ghanbarzade Miyandehi (2013), although they initially proposed a negative relationship between the two variables; their results showed a non-significant relationship between the two constructs.

On the other hand, the previous research of Areiza-Padilla (2021), also initially proposed a negative relationship between the animosity of the consumer and the image of a foreign brand, however, their results came out opposite, that is to say that animosity does not always negatively influence foreign brands, when these brands are very well positioned, in the mind of the consumer.

Taking into account the above, for this research we propose that when a foreign brand has a previous positioning in the minds of consumers in a positive way, these values will not change despite the animosity felt against the country from which the brand originates. For this reason we propose the following Hypothesis:

Hypothesis 5 (H5). Consumer animosity does not affect the image of foreign brands, as long as these brands have a positive positioning in advance

On the other hand, a distinction has been made between patriotism and nationalism, where patriotism generates strong ties and loyalty to one's own group, but without corresponding hostility to other groups, unlike nationalists, in this way the patriotic have a more cooperative and peaceful approach, as well as an approach to the world, which allows them to take pride in their own country, while recognizing its shortcomings, and even to be willing to cooperate with other nations, and incorporate them into their group (Druckman 1994).

Previous studies by Bartikowski et al. (2020), show that patriotic sentiments do not necessarily generate negative feelings towards foreign brands that represent luxury, social status and even cultural proximity. Even for authors like Chang et al. (2020), when foreign brands promote local brands at major events, they receive greater acceptance by the local consumer, in this way, it is evident that patriotism can generate positive feelings for both domestic and foreign brands.

In view of the above, we formulate the following Hypothesis:

Hypothesis 6 (H6). The patriotic tendencies of Colombian society can have a positive impact on the image of foreign brands, as long as these brands do not generate threats for Colombia

Otherwise, although some previous studies such as those of Fakharmanesh and Ghanbarzade Miyandehi (2013), Shu et al. (2013), Correa and Parente-Laverde (2017) have determined that ethnocentrism is usually positively related to the image of the country and local brands, and negatively with the image of foreign countries and products, due to the specific characteristics of ethnocentric societies which prefer their products over any foreign brand; other studies such as those by Chaudhry et al. (2020), have managed to determine that societies could be ethnocentric, but at the same time generate loyalty and a positive image towards a foreign brand.

This is because, in some cases, the brand or the product end up generating a counterweight to ethnocentrism, because its positioning is so important in the mind of the consumer, that it can generate acceptance and desirability even of an ethnocentric consumer, because, in some cases, they are perceived as a brand or product with higher quality, greater prestige, with greater functional and symbolic benefits or because their consumption is already very common.

Hypothesis 7 (H7). Ethnocentric tendencies do not affect the image of foreign brands, as long as these brands do not create threats for Colombia

### 2.4 Research model

**Figure 1:**
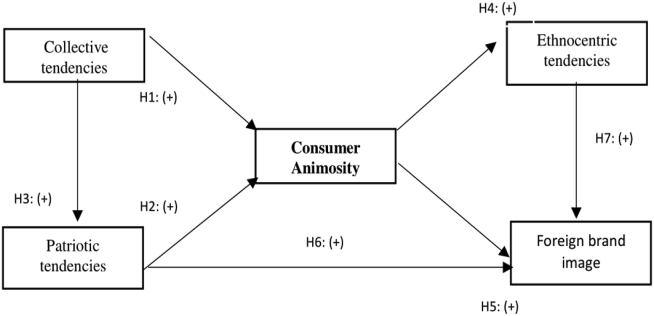
Theoretical model and Hypothesis. Source: Author's own compilation.

Figure 1 shows the research model of the present study.

## 3 Methodology

### 3.1 Data collection and sampling

To verify the proposed hypotheses, a structured online questionnaire was made to Colombian customers of the “Farmatodo” pharmacies. Participants were previously explained the general purpose of the research, the procedure, the voluntary nature of participation, and the right to stop answering the questionnaires at any time, and your consent. Similarly, they were told that the answers would be completely confidential and anonymous, because the form did not contain their personal data. All the ethical guidelines for data collection, informed consent and pertinent disclaimers were reviewed and approved by the ethics committee of Universidad Javeriana with code FCEA-DF-0222-2020. All participants were informed before the survey that the pharmacy was of Venezuelan origin, in case they did not know this data. A final sample of 298 valid questionnaires was obtained. The sample was collected in the second half of 2020. The profile of the respondents was mostly university students (45%), followed by active workers (37); of whom (53%) were women and (47%) were men. The majority of respondents were between 18 and 35 years of age (81%).

The estimation of the model and the verification of the hypotheses, was carried out through the statistical program PLS 3.2.7 for structural equations of partial least squares, which is usually used in this type of predictive studies (Barroso et al., 2010).

### 3.2 Measurement of the variables

Scales previously validated in the literature in this way were used for this research: For consumer animosity the scale of Hoffmann et al. (2011), for patriotic tendencies, Levinson (1950), and for collective tendencies, was used Triandis and Gelfand (1998). On the other hand, the scale proposed by Sharma (2015) was used to measure ethnocentric trends, while the scale of (Palacios-Florencio et al., 2018) was used to measure the image. Because the scales were originally in English, they were adapted to our object of study and translated into Spanish, retaining their grammatical meaning.

The 7-point Likert scale was used in this way: 1 = “totally disagree” and 7 = “totally agree”.

## 4 Analysis and discussion of the results

The analysis of the results was carried out in two phases. First, the possible dimensions of the variables consumer animosity, collective tendencies and ethnocentric tendencies were analyzed through an Exploratory Factor Analysis, using the SPSS program. For this research a factorial analysis was developed, since it was wanted to verify, that the items that we use to measure the variables that had several dimensions, were also grouped in the same way, as in the original work of the author. Secondly, the measuring instrument was validated by means of a Confirmatory Factor Analysis and the proposed structural model was estimated by means of Partial Least Squares Minima (PLS-SEM), using the Smart PLS 3.2.7 software. The reasons for the use of PLS-SEM were based on the fact that this study uses Mode B composite models (Henseler, 2017), on the other hand, the research model has direct and mediated relationships, It also has several levels of dimensionality with first and second order constructs (Ali et al., 2018).

### 4.1 Exploratory Factor Analysis

To determine whether the dimensions used in this paper that mediate the variables consumer animosity, collective tendencies and ethnocentric tendencies are the same as those considered in previous studies, an Exploratory Factor Analysis with VARIMAX rotation has been carried out. This determines which items should measure each dimension and whether the items are grouped in the way, they were initially proposed.

Thus, first, it was found that the items used to measure the consumer animosity construct are grouped into three factors, which, according to the semantic content of the items collected in each of them, have been called: perceived threat (ANI-PT), antithetical political attitudes (ANI-APA) and negative personal experiences (ANI-NPE), jointly explaining 89% of the variability of the phenomenon, providing a satisfactory adjustment.

Replicating the exploratory analysis on the scale used to measure the variable collective tendencies, three factors have been identified that, given the semantic content of the items they load in each of them, two dimensions have been labeled horizontal collectivism (COL-HC), with 4 items, and vertical collectivism (COL-VC), with 4 items. The two dimensions account for 75% of the variability of the phenomenon, providing a satisfactory adjustment.

For the ethnocentric trend variable, 23 items are retained, from which three factors emerge that have been called: affective reaction (SET-AR), cognitive bias (SET-CB) and behavioral preference (SET-BP), and that jointly explain 83% of the phenomenon, providing a satisfactory adjustment.

Table 2 shows the results of the adequacy measurement index of the KMO sample and the Bartlett sphericity test for the corresponding analyses.

**Table 2:** Summary of exploratory factor analysis.

Construct	KMO	Bartlett's test (Chi-square value)	Significance	Dimensions
Consumer animosity	0.779	1,064.46	0.000	3
Collective tendencies	0.813	970.64	0.000	2
Ethnocentric tendencies	0.945	5,289.76	0.000	3

Source: Author's own compilation.

For our study, we decided to use the proposed 24 items Sharma (2015) divided into 3 dimensions.

In this way we used the 8 items for the first dimension “affective reaction”, 8 items for the second dimension “cognitive bias” and the 8 items for the third dimension “behavioral preference”.

However, in our study we had to remove 1 item from the third dimension “behavioral preference” for having a load less than 0.5. Eliminating this item significantly improved the overall results of α = Cronbach's Alpha; CR = Composite reliability; and AVE = Average Variance Extracted.

For this reason, at the end we use only 23 items of this scale.

### 4.2 Confirmatory Factor Analysis (AFC)

For the evaluation of the Confirmatory Factor Analysis, in the measurement models reflective compounds (Mode A), and to analyze the reliability of the first order constructs, the individual reliability of the item (α of Cronbach) and the measure of composite reliability (CR) were analyzed.

With respect to convergent validity, all indicator loads were significant and greater than 0.7 (except for one item of the vertical variable collectivism tendencies and four items of the variable patriotism tendencies that were eliminated).

In addition, the mean extracted variance (AVE) value of each variable was greater than 0.5, providing evidence of adequate convergent validity in the measurement model (Fornell and Larcker, 1981). Table 3 shows the results of the AFC.

**Table 3:** Measurement model evaluation results.

Construct/Indicators	Mean	St.dev.	Loadings factor
**F1. Consumer animosity - perceived threat (ANI-PT) (α= 0.719; CR = 0.877; AVE = 0.780)**			
I feel threatened by Venezuela	4.86	1.95	0.906
The influence of politicians from Venezuela on our country is too strong	3.49	1.90	0.913
Venezuela intends to dominate our country economically	3.61	1.88	0.821
**F2. Consumer animosity - antithetical political attitudes (ANI-APA) (α = 0.654; CR = 0.849; AVE = 0.738)**			
I disapprove of the politics of Venezuela	4.10	1.81	0.808
I often disagree with the political attitude of Venezuela	3.85	1.83	0.907
**F3. Consumer animosity - negative personal experiences (ANI-NPE) (α = 0.704; CR = 0.811; AVE = 0.610)**			
Personally, I have had bad experiences with Venezuela	5.44	1.72	0.877
So far, I met only a few sympathetic persons from Venezuela	5.24	1.79	0.890
**F4. Horizontal collectivism (COL-HC) (α = 0.822; CR = 0.880; AVE = 0.650)**			
If a coworker gets a prize, I would feel proud	5.69	1.31	0.797
The well-being of my coworkers is important to me	5.79	1.16	0.865
To me, pleasure is spending time with others.	5.22	1.52	0.689
I feel good when I cooperate with others	5.84	1.19	0.860
**F5. Vertical collectivism (COL-VC) (α = 0.798; CR = 0.843; AVE = 0.584)**			
Parents and children must stay together as much as possible.	5.38	1.46	0.827
It is my duty to take care of my family, even when 1 have to sacrifice what I want.	5.44	1.43	0.743
It is my duty to take care of my family, even when 1 have to sacrifice what I want.	4.97	1.63	0.923
**F6. Patriotism tendencies (α = 0.834; CR = 0.882; AVE = 0.600)**			
Minor forms of military training, obedience, and discipline, such as drill, marching and simple commands, should be made a part of the elementary school educational program.	3.63	1.84	0.761
The main threat to basic Colombian institutions during this century has come from the infiltration of foreign ideas, doctrines, and agitators.	3.72	1.91	0.766
Present treatment of conscientious objectors, draft evaders, and enemy aliens is too lenient and mollycoddling. If a person won't fight for his country, he deserves a lot worse than just prison or a work camp.	3.21	1.92	0.822
In view of the present national emergency, it is highly important to limit responsible government jobs to native, white, Christian Colombians	2.89	1.82	0.795
Foreigner's refugees may need them, but it would be a big mistake to lower your immigration quotas and allow them to flood the country.	3.84	1.97	0.726
**F7. Ethnocentrism-affective reaction (SET-AR) (α = 0.0.946; CR = 0.954; AVE = 0.723)**			
I love the services from Colombia.	4.77	1.38	0.731
I am proud of the services from Colombia.	4.54	1.52	0.865
I admire the services from Colombia.	4.51	1.46	0.821
I feel attached to the services from Colombia	4.36	1.53	0.867
I hate the services from foreign countries.	4.19	1.58	0.885
I despise the services from foreign countries.	4.73	1.51	0.857
I am embarrassed by the services from foreign countries.	4.44	1.68	0.898
I feel no attachment with the services from foreign countries	4.18	1.71	0.865
**F8. Ethnocentrism-cognitive bias (SET-CB) (α = 0.886; CR = 0.954; AVE = 0.723)**			
East or West, the services from Colombia are the best.	3.97	1.59	0,798
Services from Colombia are examples of best workmanship.	4.19	1.66	0.744
Service providers from Colombia have the best work attitudes.	4.34	1.49	0.774
Products and services from foreign countries are no match for those from Colombia	3.53	1.71	0.756
Colombia has the hardest working people in the services sector.	4.66	1.49	0.717
Service providers from Colombia are more caring than those in any foreign country.	3.99	1.43	0.844
Services from Colombia are guaranteed for best performance	4.11	1.37	0.812
Colombia provides the most pleasant service experience.	4.31	1.66	0.841
**F9. Ethnocentrism-behavioural preference (SET-BP) (α = 0.875; CR = 0.886; AVE = 0.533)**			
For me, it's always the services from Colombia first, last and foremost	4.15	1.71	0.750
I prefer being served by service providers from Colombia.	4.73	1.52	0.736
As far as possible, I avoid buying services from foreign countries	4.71	1.57	0.728
I often refuse to buy service because it is from a foreign country	3.53	1.65	0.823
I would much rather not buy a product or service, than buy one from a foreign country	3.53	1.67	0.836
It may cost me in the long run but I support services from Colombia.	3.95	1.71	0.858
I will never regret buying a service from Colombia	4.14	1.84	0.718

Note: α = Cronbach's Alpha; CR = Composite reliability; AVE = Average Variance Extracted; ∗p < 0.01.

Source: Author's own compilation.

In order to analyze the discriminating validity in the measuring instrument, the Fornell and Larcker criteria and the ratio Heterotrait-Heteromethod-HT and Monotrait-Heteromethod-MT (HTMT) have been used. Using the Fornell and Larcker criteria, it has been verified that the square of the estimated correlation between two factors does not exceed in any case the variance extracted average of each factor (Fornell and Larcker, 1981), while for the HTMT ratio its values are less than 0.9 (Henseler et al., 2015), thus confirming the discriminating validity of the reflective constructs of the measurement model (Table 4).

**Table 4:** Discriminant validity.

	F1	F2	F3	F4	F5	F6	F7	F8	F9
F1	**0.883**	0.255	0.363	0.134	0.111	0.214	0.135	0.153	0.207
F2	0.805	**0.859**	0.317	0.167	0.184	0.254	0.131	0.173	0.190
F3	0.423	0.444	**0.781**	0.228	0.141	0.360	0.130	0.169	0.192
F4	0.083	0.130	0.190	**0.806**	0.563	0.119	0.298	0.280	0.298
F5	0.076	0.139	0.014	0.359	**0.764**	0.279	0.336	0.261	0.374
F6	0.169	0.188	0.324	0.061	0.290	**0.775**	0.265	0.308	0.409
F7	0.115	0.112	0.119	0.254	0.282	0.238	**0.850**	0.331	0.337
F8	0.138	0.151	0.151	0.208	0.197	0.252	0.649	**0.741**	0.361
F9	0.137	0.120	0.207	0.150	0.294	0.415	0.530	0.614	**0,730**

Note: On the diagonal in bold: square root of the AVE values. Below the diagonal: correlations. Above the diagonal: HTMT values.

Source: Author's own compilation.

For the variable image, when defined as formative (Mode B), its evaluation has been done at the level of the indicators by assessing the possible multicolineality, through the variance inflation factor (VIF) and the assessment of the magnitude of their weights and their significance, the results of which can be seen in Table 5.

**Table 5:** Measurement construct image.

Indicators	Mean	St.dev.	Weights	VIF	t	p-value
The interior of the pharmacy is appropriate for its category	5.32	1.39	-0.233	1.429	2.777	0.006
The location of pharmacy chain is suitable	5.19	1.50	-0.024	1.933	4.688	0.000
I can clearly distinguish the establishments of this pharmacy chain	5.31	1.38	0.006	1.732	7.631	0.000
I tend to pay attention to this pharmacy's advertising	4.82	1.52	-0.150	2.448	7.222	0.000
I tend to pay attention to the information they send me	5.38	1.53	-0.116	1.390	2.376	0.018
This pharmacy is renowned for its good social behaviour	4.33	1.74	0.132	1.364	1.988	0.047
This pharmacy chain's image fits my personality	4.43	1.71	0.166	1.446	2.243	0.025

Source: Author's own compilation.

The second-order variables were then analyzed. First, with respect to the variable called consumer animosity, being of a reflective-formative type, its measurement analysis has been carried out through the inflation variance factor (IVF) and weights, the results of which can be seen in Table 6.

**Table 6:** Measurement model of the second order construct **c**onsumer animosity.

First order factors	Weights	FIV	t	p valor
Perceived threat	-0.436	2.882	2.848	0.005
Antithetical political attitudes	0.182	2.949	3.131	0.000
Negative personal experiences	1.060	1.265	4.162	0.000

Source: Author's own compilation.

Second, regarding second-order reflective constructs (collective tendencies and ethnocentric tendencies), Table 7 shows the standardized loads of their corresponding dimensions, which are greater than 0.7 and statistically, the Cronbach α, the composite reliability measurement (CR) and extracted variance analysis (AVE), and it is evident that first order dimensions contribute statistically significantly to their corresponding second order reflective constructs.

**Table 7:** Measurement model of reflective second order constructs.

Construct	Dimensions	Loadings dimension	α	CR	AVE
Collective tendencies	Horizontal collectivism	0.781	0.848	0.908	0.767
	Vertical collectivism	0.901			
Ethnocentrism tendencies	SET-Affective reaction	0.788∗	0.817	0.887	0.724
	SET-Cognitive bias	0.876∗			
	SET-Behavioral preference	0.886∗			

α = Cronbach's Alpha; CR = Composite reliability; AVE = Average Variance Extracted; ∗p < 0.01.

Source: Author's own compilation.

### 4.3 Structural model and hypotheses testing

Once the psychometric properties of the measuring instrument were verified, the model of structural equations was estimated through partial least squares (PLS) and the bootstrapping procedure (Henseler, 2017), with 5000 sub-samples. As it can be seen in Table 8, the coefficients of the *paths* have been significant in all cases and in the sense that the hypotheses pointed out, except in the relations between collective tendencies and consumer animosity, on the one hand, and consumer animosity and image, on the other hand.

**Table 8:** Structural model results.

Hypothesis	Original Sample	t	p value	Contras
H1: Collective tendencies - **C**onsumer animosity	0.005	0.026	0.980	Not accepted
H2: Patriotism tendencies - **C**onsumer animosity	0.303	3.033	0.002	Accepted
H3: Collective tendencies - Patriotism tendencies	0.338	2.739	0.006	Accepted
H4: **C**onsumer animosity – Ethnocentrism tendencies	0.467	6.934	0.000	Accepted
H5: **C**onsumer animosity - Image	0.238	1.209	0.227	Not accepted
H6: Patriotism tendencies – Image	0.187	2.078	0.032	Accepted
H7: Ethnocentrism tendencies - Image	0.717	11.946	0.000	Accepted

Note: R^2^(Patriotism) = 0.114; R^2^(**C**onsumer animosity) = 0.393; R^2^(Ethnocentrism tendencies) = 0.428; R^2^(Image) = 0.542; Q^2^(Patriotism) = 0.243; Q^2^(**C**onsumer animosity) = 0.166; Q^2^(Ethnocentrism tendencies) = 0.216; Q^2^(Image) = 0.231.

Source: Author's own compilation.

The explanatory power of the structural model was also verified through the coefficients of determination R^2^, which indicate the amount of variance of the endogenous variables explained by the constructs and the Q^2^, which are greater than 0 (Table 8), so the model presents an adequate explanatory and predictive value and allows to evaluate the significance of the previously established causal relationships.

Since the relationship between consumer animosity and image was not significant, ethnocentrism tendencies were evaluated as a mediating variable between the two constructs. The significance of the indirect effects was estimated using the bootstrapping technique with 5000 samples and a 95% confidence interval. The results show that the indirect effect of consumer animosity on the image through ethnocentrism tendencies is significant and the mediation is total (see Table 9).

**Table 9:** Summary of mediating effect test.

	Total effect	Direct effect	Indirect effect	VAF	Mediation
**C**onsumer animosity – Ethnocentrism tendencies – Image	0.573∗	0.238	0,335∗	0.584	58.4%

∗p < 0.05 Source: Author's own compilation.

## 5 Discussion, research implications, and limitations

Bearing in mind that most studies on consumer animosity are conducted in industrialized countries and based on past conflicts, this study has sought to contribute to the limited literature on this variable in developing countries, more accurately of Latin America and the Caribbean, and that they are currently facing a political, economic and social conflict between them, as is the case of Colombia and Venezuela. In this way, this study makes a contribution to the literature of consumer animosity, focused on Latin America, and from the perspective of an international company that makes innovations in its business model especially in the pharmaceutical companies/retailers sector.

For this purpose, various social behaviors of people were analyzed through their collective, patriotic, and ethnocentric tendencies, regarding their relationship with foreign brands, whose country of origin is generating a direct animosity. For the specific case of this study, we analyzed the image that Colombians have towards the foreign brand of pharmaceutical chains of Venezuelan origin called “Farmatodo”. It is important to highlight the current social situation in Colombia, due to the arrival of more than 1,800,000 Venezuelan immigrants, which has generated great socio-economic problems.

On the other hand, Venezuela and Colombia have been antagonistic politicians, and on several occasions have threatened a military war. However, in this study we show that, although there is a current animosity, Colombians do not transmit their rejection towards Venezuelan brands automatically.

The results obtained show a positive relationship between patriotic tendencies and consumer animosity, confirming previous studies by Klein and Ettensoe (1999), Hoffmann et al. (2011), Yang et al. (2015), Al Ganideh and Elahee (2018), in which patriotic societies generate strong bonds of love to their country of origin, and therefore present great concerns when feeling an external threat through a foreign country, confirming the Hypothesis (H2).

We also find a positive relationship between collective tendencies and patriotic tendencies. In this research it had been suggested that, in societies with high values of collectivism, by carrying out constant actions that seek to foster social cohesion among the members of their group, they would end up exalting their levels of patriotism, as a sign of pride, loyalty and love to their own country. In this way, we can contribute to the previous interpretations of Realo and Allik (1999); Sagy et al. (2001); Anthony et al. (2003); Steele and Lynch (2013) and confirm the Hypothesis (H3).

Similarly, we were able to confirm the Hypothesis (H4) regarding the positive relationship between consumer animosity and ethnocentric trends, where we can see that animosity is one of the factors that directly influences ethnocentrism, because rejection towards a specific group, may end up generating in some people the rejection towards other external groups, as a mechanism of general protection towards their own social group; in this way we contribute to the previous studies of Nakos and Hajidimitriou (2007), Cheah et al. (2016), Lee et al. (2017), Souiden et al. (2018).

On the other hand, the results indicated that there was no significant relationship between collectivism and animosity, so we had to reject the Hypothesis (H1) these data were different to those presented by Han (2017); Latif et al. (2019), so in our study, we can say that the collective values of the countries of Latin America are not related to animosity.

Similarly, the initial results indicated that there is no significant relationship between animosity and the image of a foreign brand, having to reject the Hypothesis (H5).

However, it was found that by taking ethnocentric trends as a mediating variable between consumer animosity and image, a significant result is presented through such mediation, that is to say that the animosity of the consumer does not come directly to the image, but through mediation with ethnocentrism.

As a novelty, this research has shown that a person, although he may have high tendencies of patriotism, may have a positive image of a foreign brand, thus confirming the Hypothesis (H6); similarly we were able to confirm that ethnocentric tendencies, may have a positive image of foreign products confirming the Hypothesis (H7).

This can be explained, due to several factors: As indicated above, patriotic values highlight the love for your own country, but do not necessarily generate negative feelings, towards foreign brands that represent luxury, social status, cultural proximity, Bartikowski et al. (2020); Chang et al. (2020); on the other hand, previous studies by Chaudhry et al. (2020) have shown that a person can be ethnocentric, and have a loyalty and positive image towards foreign brands, which are very strong brands because they are global brands or because their consumption generates social status, or because their consumption is already daily.

On the other hand, the chain of pharmacies “Farmatodo” has been operating in Colombia for more than 20 years with 59 stores in 6 cities, so its consumption is already common on the part of Colombians, even managing to position itself in the minds of younger consumers as a local brand. Some of the younger respondents did not know that this brand was of Venezuelan origin, for this reason they were made this clarification at the beginning of the questionnaire; however, from the results obtained we can say that this factor did not negatively influence the valuation of the brand.

“Farmatodo” is a chain of pharmacies with much larger and more modern facilities than neighborhood pharmacies, and located in points of heavy commercial traffic. In addition, it not only sells exclusive products from a pharmacy, but also several different categories, which allow you to have catalog products is very wide, where a consumer can meet several different needs in one place.

It has also managed to position itself in the minds of consumers as the fastest pharmacy in online order delivery, with an average duration of 30 or 45 min. Customer delivery times are based on being able to know your inventory in real time and the triangulation of all your stores, website and mobile application.

All of this has been achieved by managing various innovations through Oracle Retail Merchandising System, Oracle Retail Store Inventory Management, Oracle Retail Warehouse Management System, and Oracle Retail Xstore Point-of-Service.

With this, we can say that although there is animosity against a particular country, it does not mean that a general animosity against its products or brands is automatically generated, especially if these brands already had a regular consumption before animosity was generated.

In this way, just as animosity is studied about a specific country, through this study we want to propose that consumer animosity should be studied about brands, specific products or services and not generally towards all products originating in the country causing the animosity.

Finally, we consider some limitations in this research, which are also possible lines of research. This study focused on the “Farmatodo” chain, however, we propose to analyze other Venezuelan companies in the manufacturing, tourism, hydrocarbons and food sector. In the same way, study companies with less than 5 years of operations in Colombia and that are not so positioned yet in the consumer's mind.

On the other hand, we consider that we should also study the quality of the online media service, focused on the design of the website, security, privacy and also on compliance with delivery times (Rita et al., 2019). Finally, for this study, the “CES” Sharma scale (2015) was used to measure ethnocentrism. We propose to replicate this study using the “CESTCALE” scale of Shimp and Sharma (1987) which is very common to measure this variable, and to be able to identify if there are differences or similarities between the results of both scales.

## Declarations

### Author contribution statement

Jose Andres Areiza-Padilla: Conceived and designed the experiments; Performed the experiments; Analyzed and interpreted the data, Contributed reagents, materials, analysis tools or data; Wrote the paper.

Mario Andres Manzi Puertas and Mihaela Simona Moise: Analyzed and interpreted the data, Contributed reagents, materials, analysis tools or data; Wrote the paper.

### Funding statement

This research did not receive any specific grant from funding agencies in the public, commercial, or not-for-profit sectors.

### Data availability statement

Data included in article/supplementary material/referenced in article.

### Declaration of interests statement

The authors declare no conflict of interest.

### Additional information

No additional information is available for this paper.

## References

[bib1] Amine L.S. (2008). Country-of-origin, animosity and consumer response: marketing implications of anti-Americanism and Francophobia. Int. Bus. Rev..

[bib2] Anthony K., Rosselli F., Caparyan L. (2003). Truly evil or simply angry: individualism, collectivism, and attributions for the events of september 11th. Indiv. Differ. Res..

[bib3] Al Ganideh S.F., Elahee M.N. (2018). Dealing with “enemy-brothers”: sunni Arab consumers’ animosity toward Iran and Turkey. J. Consum. Market..

[bib4] Ali F., Rasoolimanesh S.M., Sarstedt M., Ringle C.M., Ryu K. (2018). An assessment of the use of partial least squares structural equation modeling (PLS-SEM) in hospitality research. Int. J. Contemp. Hospit. Manag..

[bib5] Alvarez M.D., Campo S. (2020). Consumer animosity and its influence on visiting decisions of US citizens. Curr. Issues Tourism.

[bib6] Aliaga Sáez F.A., Flórez de Andrade A., García Sicard N., Díaz Medina F. (2020). La integración de los venezolanos en Colombia: discurso de líderes inmigrantes en Bogotá y Cúcuta. Sociol. - Probl. Prat..

[bib7] Areiza-Padilla J.A. (2021). Decreasing consumer animosity: the relationship between fast food businesses and social conflicts in Latin America and the Caribbean. Cogent Business Manag..

[bib8] Averill J.R. (1983). Studies on anger and aggression: implications for theories of emotion. Am. Psychol..

[bib9] Bartikowski B., Fastoso F., Gierl H. (2020). How nationalistic appeals affect foreign luxury brand reputation: a study of ambivalent effects. J. Bus. Ethics.

[bib10] Barroso C., Carrión G.C., Roldán J.L. (2010). Applying maximum likelihood and PLS on different sample sizes: studies on SERVQUAL model and employee behavior model. Handbook of Partial Least Squares.

[bib11] Baruk A.I. (2019). The effect of consumers’ ethnocentric attitudes on their willingness for prosumption. Heliyon.

[bib12] Bizumic B. (2018). Ethnocentrism: Integrated Perspectives.

[bib13] Brummett B.H., Maynard K.E., Babyak M.A., Haney T.L., Siegler I.C., Helms M.J., Barefoot J.C. (1998). Measures of hostility as predictors of facial affect during social interaction: evidence for construct validity. Ann. Behav. Med..

[bib14] Chang C.H., King B.E., Shu S.T. (2020). Tourist attitudes to mega-event sponsors: where does patriotism fit?. J. Vacat. Mark..

[bib15] Chaudhry N.I., Mughal S.a., Chaudhry J.I., Bhatti U.T. (2020). Impact of consumer ethnocentrism and animosity on brand image and brand loyalty through product judgment. J. Islam. Market..

[bib16] Cheah I., Phau I., Kea G., Huang Y.A. (2016). Modelling effects of consumer animosity: consumers' willingness to buy foreign and hybrid products. J. Retailing Consum. Serv..

[bib17] Correa S., Parente-Laverde A.M. (2017). Consumer ethnocentrism, country image and local brand preference: the case of the Colombian textile, apparel and leather industry. Global Bus. Rev..

[bib18] Doocy S., Page K.R., de la Hoz F., Spiegel P., Beyrer C. (2019). Venezuelan migration and the border health crisis in Colombia and Brazil. J. Mig. Human Secur..

[bib19] Druckman D. (1994). Nationalism, patriotism, and group loyalty: a social psychological perspective. Mershon Int. Stud. Rev..

[bib20] Durkheim É., rookfield C., Turner B.S. (2018). Professional Ethics and Civic Morals.

[bib21] Fakharmanesh S., Ghanbarzade Miyandehi R. (2013). The purchase of foreign products: the role of brand image, ethnocentrism and animosity: Iran market evidence. Iran. J. Manag. Stud..

[bib22] Fornell C., Larcker D.F. (1981). Evaluating structural equation models with unobservable variables and measurement error. J. Market. Res..

[bib23] Greif A. (1994). Cultural beliefs and the organization of society: a historical and theoretical reflection on collectivist and individualist societies. J. Polit. Econ..

[bib24] Han C.M. (2017). Individualism, collectivism, and consumer animosity in emerging Asia: evidence from Korea. J. Consum. Market..

[bib25] Henseler J., Ringle C.M., Sarstedt M. (2015). A new criterion for assessing discriminant validity in variance-based structural equation modeling. J. Acad. Market. Sci..

[bib26] Henseler J. (2017). Bridging design and behavioral research with variance-based structural equation modeling. J. Advert..

[bib27] Hoffmann S., Mai R., Smirnova M. (2011). Development and validation of a cross-nationally stable scale of consumer animosity. J. Market. Theor. Pract..

[bib28] Isakov A., Fowler J.H., Airoldi E.M., Christakis N.A. (2019). The structure of negative social ties in rural village networks. Sociolog. Sci..

[bib29] Jung K., Ang S.H., Leong S.M., Tan S.J., Pornpitakpan C., Kau A.K. (2002). A typology of animosity and its cross-national validation. J. Cross Cult. Psychol..

[bib30] Keller K.L. (1993). Conceptualizing, measuring, and managing customer-based brand equity. J. Market..

[bib31] Klasing M.J. (2013). Cultural dimensions, collective values and their importance for institutions. J. Comp. Econ..

[bib32] Klein J.G., Ettenson R., Morris M.D. (1998). The animosity model of foreign product purchase: an empirical test in the People's Republic of China. J. Market..

[bib33] Klein J.G., Ettensoe R. (1999). Consumer animosity and consumer ethnocentrism: an analysis of unique antecedents. J. Int. Consum. Market..

[bib34] Latif K., Pitafi A.H., Malik M.Y., Latif Z. (2019). Individual cultural values and consumer animosity: Chinese consumers’ attitude toward American products. Sage Open.

[bib35] Lee R., Lee K.T., Li J. (2017). A memory theory perspective of consumer ethnocentrism and animosity. Eur. J. Market..

[bib36] Levinson D.J. (1950). The study of ethnocentric ideology. Author. Personal..

[bib37] Li Y., Li B., Wang G., Yang S. (2021). The effects of consumer animosity on demand for sharing-based accommodations: evidence from Airbnb. Decis. Support Syst..

[bib38] Little J.P., Singh N. (2015). Decontextualizing consumer animosity. J. Global Market..

[bib39] Maher A.A., Clark P., Maher A. (2010). International consumer admiration and the persistence of animosity. J. Consum. Market..

[bib40] Martínez Moya D.A. (2020). The Effect of a Labor Supply Shock on Factors Productivity: the Case of a Venezuelan Migration in Colombia.

[bib41] Migracion Colombia (2020). Más de 1 millón 825 mil venezolanos estarían radicados en Colombia. https://www.migracioncolombia.gov.co/noticias/mas-de-1-millon-825-mil-venezolanos-estarian-radicados-en-colombia.

[bib42] Nakos G.E., Hajidimitriou Y.A. (2007). The impact of national animosity on consumer purchases: the modifying factor of personal characteristics. J. Int. Consum. Market..

[bib43] Nijssen E.J., Douglas S.P. (2004). Examining the animosity model in a country with a high level of foreign trade. Int. J. Res. Market..

[bib44] Palacios-Florencio B., García del Junco J., Castellanos-Verdugo M., Rosa-Díaz I.M. (2018). Trust as mediator of corporate social responsibility, image and loyalty in the hotel sector. J. Sustain. Tourism.

[bib45] Realo A., Allik J. (1999). A cross-cultural study of collectivism: a comparison of American, Estonian, and Russian students. J. Soc. Psychol..

[bib46] Rita P., Oliveira T., Farisa A. (2019). The impact of e-service quality and customer satisfaction on customer behavior in online shopping. Heliyon.

[bib47] Rose M., Rose G.M., Shoham A. (2009). The impact of consumer animosity on attitudes towards foreign goods: a study of Jewish and Arab Israelis. J. Consum. Market..

[bib48] Roth M.S. (1995). The effects of culture and socioeconomics on the performance of global brand image strategies. J. Market. Res..

[bib49] Russell C.A., Russell D.W. (2010). Guilty by stereotypic association: country animosity and brand prejudice and discrimination. Market. Lett..

[bib50] Sagy S., Orr E., Bar-On D., Awwad E. (2001). Individualism and collectivism in two conflicted societies: comparing Israeli-Jewish and Palestinian-Arab high school students. Youth Soc..

[bib51] Salinas E.M., Pérez J.M.P. (2009). Modeling the brand extensions' influence on brand image. J. Bus. Res..

[bib52] Schopmeyer K.D., Fisher B.J. (1993). Insiders and outsiders: exploring ethnocentrism and cultural relativity in sociology courses. Teach. Sociol..

[bib53] Sharma P. (2015). Consumer ethnocentrism: reconceptualization and cross-cultural validation. J. Int. Bus. Stud..

[bib63] Shimp T.A., Sharma S. (1987). Consumer ethnocentrism: construction and validation of the CETSCALE. J. Market. Res..

[bib54] Souiden N., Ladhari R., Chang L. (2018). Chinese perception and willingness to buy Taiwanese brands: the role of ethnocentrism and animosity. Asia Pac. J. Market. Logist..

[bib55] Shu S.T., Strombeck S., Hsieh C.L. (2013). Consumer ethnocentrism, self-image congruence and local brand preference: a cross-national examination. Asia Pac. Manag. Rev..

[bib56] Steele L.G., Lynch S.M. (2013). The pursuit of happiness in China: individualism, collectivism, and subjective well-being during China’s economic and social transformation. Soc. Indicat. Res..

[bib57] Sumner W.G. (1906). Folkways: A Study of the Sociological Importance of Usages, Manners, Customs, Mores, and Morals.

[bib58] Triandis H.C. (2018). Individualism and Collectivism.

[bib59] Triandis H.C., Gelfand M.J. (1998). Converging measurement of horizontal and vertical individualism and collectivism. J. Pers. Soc. Psychol..

[bib60] Triandis H.C., McCusker C., Hui C.H. (1990). Multimethod probes of individualism and collectivism. J. Pers. Soc. Psychol..

[bib61] Van der Toorn J., Nail P.R., Liviatan I., Jost J.T. (2014). My country, right or wrong: does activating system justification motivation eliminate the liberal-conservative gap in patriotism?. J. Exp. Soc. Psychol..

[bib62] Yang Q., Snell K., Tsai W.S. (2015). Understanding consumer animosity in the politicized global market: from the perspective of young transnational consumers. J. Int. Consum. Market..

